# Biocontrol of plant parasitic nematodes by bacteria and fungi: a multi-omics approach for the exploration of novel nematicides in sustainable agriculture

**DOI:** 10.3389/fmicb.2024.1433716

**Published:** 2024-07-26

**Authors:** Muhammad Ayaz, Jing-Tian Zhao, Wei Zhao, Yuan-Kai Chi, Qurban Ali, Farman Ali, Abdur Rashid Khan, Qing Yu, Jing-Wen Yu, Wen-Cui Wu, Ren-De Qi, Wen-Kun Huang

**Affiliations:** ^1^State Key Laboratory for Biology of Plant Diseases and Insect Pests, Institute of Plant Protection, Chinese Academy of Agricultural Sciences, Beijing, China; ^2^Institute of Plant Protection and Agro-Products Safety, Anhui Academy of Agricultural Sciences, Hefei, China; ^3^Department of Biology, College of Science, United Arab Emirates University, Al Ain, United Arab Emirates; ^4^Department of Entomology, Abdul Wali Khan University Mardan, Mardan, Pakistan; ^5^Key Laboratory of Integrated Management of Crop Diseases and Pests, Department of Plant Pathology, College of Plant Protection, Nanjing Agricultural University, Nanjing, China

**Keywords:** biocontrol agents, plant parasitic nematodes, multi-omics approaches, novel nematicides, sustainable agriculture

## Abstract

Plant parasitic nematodes (PPNs) pose a significant threat to global crop productivity, causing an estimated annual loss of US $157 billion in the agriculture industry. While synthetic chemical nematicides can effectively control PPNs, their overuse has detrimental effects on human health and the environment. Biocontrol agents (BCAs), such as bacteria and fungi in the rhizosphere, are safe and promising alternatives for PPNs control. These BCAs interact with plant roots and produce extracellular enzymes, secondary metabolites, toxins, and volatile organic compounds (VOCs) to suppress nematodes. Plant root exudates also play a crucial role in attracting beneficial microbes toward infested roots. The complex interaction between plants and microbes in the rhizosphere against PPNs is mostly untapped which opens new avenues for discovering novel nematicides through multi-omics techniques. Advanced omics approaches, including metagenomics, transcriptomics, proteomics, and metabolomics, have led to the discovery of nematicidal compounds. This review summarizes the status of bacterial and fungal biocontrol strategies and their mechanisms for PPNs control. The importance of omics-based approaches for the exploration of novel nematicides and future directions in the biocontrol of PPNs are also addressed. The review highlighted the potential significance of multi-omics techniques in biocontrol of PPNs to ensure sustainable agriculture.

## 1 Introduction

Plant parasitic nematodes (PPNs) pose a major worldwide threat to important agricultural crop productivity. There are over 4,100 documented species of PPNs (Nicol et al., 2011), including cyst nematodes (*Heterodera* and *Globodera* spp.), root-knot nematodes (*Meloidogyne* spp.), lesion nematodes (*Pratylenchus* spp.), and dagger nematodes (*Xiphinema* spp.). It is estimated that PPNs cause an average yield loss of 12.6% equal to $157 billion in 20 major commercial crops including tomato, soybean, peanut, potato, rice and sugarcane around the world (Ayaz et al., 2021; Ning et al., 2022). They infect a wide range of plants, impacting their health and overall productivity. Root Knot nematodes (RKNs) species such as *M. javanica*, *M. incognita*, *M. hapla*, and *M. arenaria* can damage important crops by up to 90% and increase their susceptibility to other pathogens. Being root invaders, they disrupt nutrient and water uptake, leading to reduced crop growth and yield (Migunova and Sasanelli, 2021). PPNs weaken plant defense, making them more susceptible to other diseases. They secrete effector proteins and affect the host plants’ defensive mechanisms, which results in a significant economic impact on agricultural production (Ayaz et al., 2021; Ali et al., 2023b).

PPNs mostly dwell in soil and affect all parts of plants like roots, stems, leaves, and flowers. They feed by penetrating the plant cells with a flexible style. The stylet is attached to three or five pharyngeal glands, helping in penetration, effectors delivery, and parasitism (Kishore et al., 2022). The PPNs can be divided into two groups based on their feeding behaviors such as ectoparasitic and endoparasitic. However, the most common notaries and damaging sedentary endoparasitic are root-knot nematodes and cyst nematodes. The global growing population needs more food, therefore, the control of PPNs in different regions of the world requires more attention to sustainable agriculture (Pires et al., 2022; Mokrini et al., 2024).

The most common and effective method for controlling PPNs is using synthetic nematicides. However, the excessive use of agrochemicals poses negative effects on human health and the environment, leading to legislative pressure and a ban on these chemicals. This has prompted the development of alternative strategies for PPN control (Gao et al., 2016; Ali et al., 2023b). BCAs offer a promising alternative and environmental approach for managing PPNs cost-effectively (Ali et al., 2023a). The BCAs play important role in plant growth promotion under stress condition. Various rhizospheric bacteria and fungi, such as *Bacillus thuringiensis, Xenorhabdus bovienii*, *Pseudomonas fluorescens, B. amyloliquefaciens, Pasteuria penetrans*, *Trichoderma harzianum*, and *Paecilomyces lilacinus* have been found to effectively control PPNs through different mechanisms, including preventing egg hatching, destroying females and second-stage juvenile nematodes (J2s) in the soil (Kishore et al., 2022; Lawal et al., 2022). These BCAs can directly or indirectly combat PPNs by competing for nutrients and niche, producing lytic enzymes, antibiotics, volatile compounds, and toxic metabolites. Indirect antagonism occurs through the induction of plant ISR or the release of small molecules in the rhizosphere that affect nematodes feeding behavior and sex ratio (Ayaz et al., 2021; Migunova and Sasanelli, 2021).

Many plants growth-promoting rhizobacteria (PGPR) and beneficial fungi enhance plant health by triggering ISR, which boost plant defense mechanisms against different phytopathogens (Song et al., 2022). However, the knowledge regarding the complex communication network between plants and microbes in the rhizosphere against nematodes is still limited (Engelbrecht et al., 2018). In the current omics era, advanced technologies, and approaches, such as metabolomics, offer new opportunities for developing novel nematicides to improve biocontrol strategies. By using omics techniques, particularly metabolomics, with high throughput analysis novel nematicides can be developed to efficiently control PPNs (White et al., 2017; Mohanram Praveen, 2019). The objectives of the present review are to summarize the use of bacterial and fungal biocontrol agents for controlling plant PPNs and emphasize the importance of omics-based biocontrol of nematodes for discovering novel nematicides in the future. The review will also discuss the challenges and future directions for the biocontrol of PPNs to ensure sustainable agriculture.

## 2 Bacterial biocontrol agents for PPNs

Nematodes and microbial flora are two crucial elements of the soil biotic ecosystem that have coevolved over long periods. Utilizing bacteria as biological control agents to suppress nematodes in the plant rhizosphere ensures efficient control of PPNs (AbdelRazek and Yaseen, 2020; Sun et al., 2021). The influence of plant growth-promoting bacteria (PGPB) on PPNs population density is particularly significant, with genera such as *Pseudomonas, Serratia,* and *Bacillus*, showing high levels of biological control efficiency during the previous two decades (Khabbaz et al., 2019; Ayaz et al., 2021). The *Bacillus* spp. strains have been reported to produce a wide range of secondary metabolites and volatile organic compounds (VOCs) with strong nematicidal activity against PPNs (Seong et al., 2021; Ali et al., 2023a).

Studies have shown that *B. subtilis* OKB105 and *B. cereus* 09B18 culture filtrates possess strong nematicidal activity up to 95% against J2s of *M. javanica* and *H. filipjevi* (Xia et al., 2011; Zhang et al., 2016). The endophytic bacterium K6, isolated from coffee plant leaves, caused 65% mortality of *P. coffea* while *B. subtilis* OKB105 and *B. amyloliquefaciens* B3 showed nematicidal activity against *Aphelenchoides besseyi*, *Ditylenchus destructor*, and *Bursaphelenchus xylophilus* nematodes with percent mortalities of 85, 79, and 100%, respectively. The report also showed that *Serratia plymuthica* M24T3 also exhibited a strong nematicidal activity with a 100% mortality rate against pinewood (Proença et al., 2019; Migunova and Sasanelli, 2021). However, some PGPBs are effective in promoting plant growth but not in suppressing plant nematodes as reported by numerous studies conducted in a greenhouse, microplate, and field conditions (Ayaz et al., 2021; Ali et al., 2023b). *Serratia proteamaculans* Sneb 851 has shown high nematicidal potential against *M. incognita*, while *Bacillus thuringiensis* strains are known for their inhibitory potential against PPNs through *Cry* protein production (Jisha et al., 2013; Zhao et al., 2018).

*Pseudomonas fluorescens* F113 has strongly affected *G. rostochiensis* J2 stage nematodes mobility and egg hatchability. *B. firmus* GB-126 was also reported to control *R. reniformis* involving egg hatching reduction and alleviation of infestation in cotton (Cronin et al., 1997; Castillo et al., 2013)*. Bacillus* spp. strains have also been reported to produce volatile organic compounds (VOCs) with strong nematicidal activity. For example, *B. atrophaeus* GBSC56 VOCs such as dimethyl disulfide (DMDS), 2-undecanone (2-UD) and methyl isovalerate (MIV) demonstrated strong nematicidal activity up to 90% against *M. incognita*. These VOCs induced severe oxidative stress in nematodes, which subsequently caused rapid death. It also promoted plant growth and triggered ISR against *M. incognita* in both *in vitro* and in greenhouse experiments (Ayaz et al., 2021). The most effective PGPB against different PPNs reported in the literature were summarized in Table 1.

**Table 1:** Bacterial biocontrol agents against different plant parasitic nematodes.

Plant	Bacterial biocontrol agents	Plant nematodes	Mode of action	References
Tomato	*Bacillus atrophaeus* GBSC56	*Meloidogyne incognita*	ROS induction in nematodes, stimulates plant growth and defense mechanism	Ayaz et al. (2021)
Rice	*Pseudomonas simiae* MB751	*Meloidogyne incognita*	Induced systemic resistance, plant growth promotion, and *M. incognita* suppression	Sun et al. (2021)
Chili pepper	*Serratia ureilytica*	*Nacobbus aberran*	Significantly reduced eggs number, root galls., and nematode reproduction	Wong-Villarreal et al. (2021)
Potato	*Pseudomonas fluorescens* F113	*Globodera rostochiensis*	Strongly affects juvenile mobility and egg hatchability	Cronin et al. (1997)
Tomato	*Serratia plymuthica* Sneb2001	*Meloidogyne incognita*	J2 stage nematodes mortality and reduced egg hatching	Zhao et al. (2021)
Cotton	*Bacillus firmus* GB-126	*Rotylenchulus reniformis*	Nematodes egg hatching reduction and alleviation of infestation in cotton	Castillo et al. (2013)
Rice	*Bacillus thuringiensis* GBAC46	*Meloidogyne incognita*	Antagonistic activity through different proteins (*Cry31Aa* and *Cry41ORF*)	Liang et al. (2022)
Pepper	*Burkholderia cepacia*	*Meloidogyne incognita*	The reduction in nematode population was observed in pepper and a 60% inhibition rate was noticed *in vitro*	Meyer et al. (2000)
Rice	*Bacillus* spp. GBSC56, SYST2, and FZB42	*Aphelenchoides besseyi*	The VOCs of *Bacillus* spp. promoted rice growth and strongly killed the nematodes by severe oxidative stress induction	Ali et al. (2023b)
Pine trees	*Bacillus thuringiensis*	*Bursaphelenchus xylophilus*	*Cry* proteins result in vacuolization, intestinal wall contraction, thinning and shrinkage	Guo et al. (2022)
Cotton and Soybean	*Pasteuria* sp. Ph3	*Rotylenchulus reniformis*	The nematodes egg hatching inhibition and infestation reduction in cotton, soybean, and vegetables	Abd-Elgawad (2024)
Tomato	*Paenibacillus polymyxa* KM2501-1	*Meloidogyne* spp.	The use of “honey traps” to attract *M. incognita* and eliminate it by contact or fumigation	Cheng et al. (2017)
Tomato and cucumber	*B. firmus* I-1582	*Meloidogyne* spp.	Root colonization. ISR induction, egg-hatching reduction and killing of nematodes	Ghahremani et al. (2020)
Tomato	*Bacillus velezensis* YS-AT-DS1	*Meloidogyne incognita*	Significantly reduced the number of galls and egg masses on tomato roots, as well as the J2 stage nematodes infection	Hu et al. (2022)
Tomato	*Bacillus halotolerans* LYSX1	*Meloidogyne javanica*	Biocontrol of nematodes in pot experiment through ISR induction and regulation of defense linked genes	Xia et al. (2019)
Onion	*Streptomyces microflavus* A12	*Pratylenchus penetrans*	The ability to produce lytic enzymes, such as proteases and chitinases, and form biofilms necessary for colonizing the rhizosphere of plants	Marin-Bruzos et al. (2021)
Rice	*Pseudomonas rhodesiae* GC-7	*Meloidogyne graminicola*	Inhibition of egg hatching, decrease in gall index and nematode population in soil, and induction of ISR in rice plants to combat nematode infestation	Ye et al. (2022)

## 3 Fungal biocontrol agents for PPNs

The fungal biocontrol agents to control PPNs is a topic of great interest among researchers. It has been discovered that fungi and their metabolites effectively suppress the PPNs population in agriculturally important crops. The fungi are believed to be a major source of bioactive compounds to control PPNs. Fungal biocontrol of PPNs is an area of active research that aims to develop strategies for combating PPNs (Tariq et al., 2018; Pires et al., 2022). *Trichoderma* species are well known for their ability to parasitize infectious juveniles to prevent nematodes from entering the roots, thereby enhancing crop growth and productivity (Herrera-Parra et al., 2018; Mukhtar et al., 2021). The RKNs in tomatoes were significantly inhibited by *Trichoderma* strain TH by enhancing phenols, flavonoids, lignin, cellulose, jasmonic acid (JA), and salicylic acid (SA), and decreased the levels of hydrogen peroxide (H_2_O_2_), malondialdehyde (MDA), and electrolyte leakage (Yan et al., 2021). *T. asperellum* T00 regulates defense enzymes and has enormous potential as a biocontrol agent against *P. brachyurus* in soybean plants. Studies also showed that *T. harzianum* could efficiently inhibit *M. incognita* infection with a 61.88% reduction rate in tomato plants. It was also reported that *T. harzianum* reduced the amount of nematode population, egg masses, and root gall index in brinjal plants. Other studies showed that tomatoes developed systemic resistance against the RKN by *P. chlamydosporia* M10.43.2 involving reduced infection (32–43%) and female reproduction (14.7–27.6%) (Dababat and Sikora, 2007; Ghahremani et al., 2019). The endophytic fungus *Acremonium sclerotigenum* was also observed to increase *M. incognita* J2s mortality (95%) and decrease egg-hatching rates (Yao et al., 2023). A novel endophytic fungus *Chaetomium ascotrichoides* 1–24-2 from *Pinus massoniana* was found effective against *B. xylophilus with* 99% mortality rate and reduced the nematode infestation in pine seedling (Kamaruzzaman et al., 2024).

Nematophagous fungi employ various mechanisms to target nematodes, including predatory fungi like *Arthrobotrys oligospora* and *Drechslerella* spp., which form large constricting rings and hyphal networks to trap nematodes. Endoparasitic fungi, an obligatory parasite of nematodes, such as *Drechmeria coniospora*, directly attach to nematodes to kill them. Facultative parasitic fungi, such as *Pochonia chlamydosporia*, *Paecilomyces lilacinus*, and *Pochonia rubescens* target sedentary nematode stages, including nematode eggs, cysts, as well as adult female (Al-Ani et al., 2022; Kishore et al., 2022). Some nematophagous fungi can also function as facultative saprotrophs, feeding on organic matter in the absence of nematodes. Soil rich in organic matter supports the survival of these fungi. There is yet another group of toxin-producing fungi, such as *Pleurotus ostreatus* that immobilize nematodes before penetrating their cuticles with hyphae (Pires et al., 2022; de Freitas Soares et al., 2023). Various fungal biocontrol agents have been identified for targeting different types of nematodes, as summarized in Table 2.

**Table 2:** Fungal biocontrol agents against different plant parasitic nematodes.

Plant	Fungal biocontrol agents	Plant nematodes	Mode of action	References
Rice	*Volutella citrinella*	*Aphelenchoides besseyi*	The hyphae of *V. citrinella* exhibited nematicidal and predatory activity by producing rings of various sizes	Zhang et al. (2021)
Pineapple	*Purpureocillium lilacinum*	*Meloidogyne javanica*	The use of *P. lilacinum* greatly decreased the formation of nematode eggs and egg masses, minimizing the damage caused by root galling in pineapple	Kiriga et al. (2018)
Carrot	*Pochonia chlamydosporia*	*Meloidogyne incognita*	*P. chlamydosporia* decreased nematode galls and second-stage juveniles	Bontempo et al. (2014)
Apple	*Verticillium leptobactrum*	*Pratylenchus vulnus*	The population of *P. vulnus* in the soil or the roots of plants inoculated with nematophagous fungi was typically significantly reduced	Noura et al. (2018)
Tomato	*Pochonia chlamydospori* M10.43.21	*Meloidogyne incognita*	Tomatoes developed systemic resistance against the RKN by M10.43.21, which also reduced infection (32–43%) and female reproduction (14.7–27.6%)	Ghahremani et al. (2019)
Sweet corn	*Purpureocillium lilacinum*	*Heterodera zeae*	Applying *P. lilacinus* along with neem cake and karanj leaves together led to a 63.04 and 52.17% decrease in the cyst population in the soil, respectively	Baheti et al. (2015)
Rice	*Acremonium sclerotigenum*	*Meloidogyne incognita*	*M. incognita* juvenile (J2 stage) mortality is increased by *A. sclerotigenum*, whereas egg-hatching rates are decreased. Greatly lowered the galling index and suppressed the population of root-knot nematodes	Yao et al. (2023)
Brinjal	*Trichoderma harzianum*	*Meloidogyne incognita*	*T. harzianum* reduced the amount of nematode population, egg masses, and root galling in the soil	Dababat and Sikora (2007)
Soybean	*Trichoderma asperellum* T00	*Pratylenchus brachyurus*	*T. asperellum* T00 regulates defense enzymes and has enormous potential as a biocontrol agent against *P. brachyurus* in soybean plants	de Oliveira et al. (2021)
Rice	*Aspergillus welwitschia*	*Meloidogyne graminicola*	Decreased nematode attraction to rice roots, inhibited nematode growth and infection in greenhouse conditions	Xiang et al. (2020)
Tomato	*Lecanicillium muscarium*	*Meloidogyne incognita*	Nematode eggs parasitization, second-stage female juveniles	Hussain et al. (2018)
Tomato	*Mortierella globalpina*	*Meloidogyne chitwoodi*	Hyphae-mediated adherence, entrapment, and penetration of the nematode cuticle	DiLegge et al. (2019)
Mung bean	*Purpureocillium lilacinum*	*Meloidogyne incognita*	Affect the life cycle of nematodes to reduce infestation	El-Ashry et al. (2021)
Wheat	*Beauveria bassiana* 08F04	*Heterodera filipjevi*	The fungus reduces *H*. *filipjevi* females in roots by up to 64.4% through effective colonization	Zhang et al. (2020)
Tomato	*Trichoderma harzianums* TH	*Meloidogyne incognita*	With a 61.88% RKN reduction rate in tomato plants*, T. harzianum* (TH) could efficiently inhibit *M. incognita* infection	Yan et al. (2021)
Tomato	*Arthrobotrys oligospora*	*Meloidogyne incognita*	Development of constrictive rings to trap nematodes	Soliman et al. (2021)
Cucumber	*Penicillium chrysogenum* Snef1216	*Meloidogyne incognita*	Nematode reproduction is directly inhibited, reduce eggs hatching	Sikandar et al. (2019)

## 4 Biocontrol mechanisms of PPNs suppression

BCAs, particularly bacteria and fungi employ various strategies to suppress PPNs population in both laboratory and field experiments. The BCAs are mostly documented to suppress PPNs directly or indirectly through different methods (Ayaz et al., 2021; Liang et al., 2022). Bacterial BCAs produce a wide variety of products, including extracellular enzymes, toxins, secondary metabolites, and VOCs to kill nematodes directly or induce plant ISR induction for indirect PPN suppression by regulating defense-related genes (Migunova and Sasanelli, 2021; Ali et al., 2023b). PGPR competes for nutrients and space, affecting nematode populations in the rhizosphere. Fungal BCAs also exhibit strong nematicidal activity through the production of bioactive compounds that can kill nematodes directly or induce ISR against nematodes by regulating defense-related genes (Gao et al., 2016; Pires et al., 2022). The mechanisms of bacterial and fungal for PPN control are briefly discussed below.

### 4.1 Bacterial biocontrol mechanisms for PPNs

**Figure 1:**
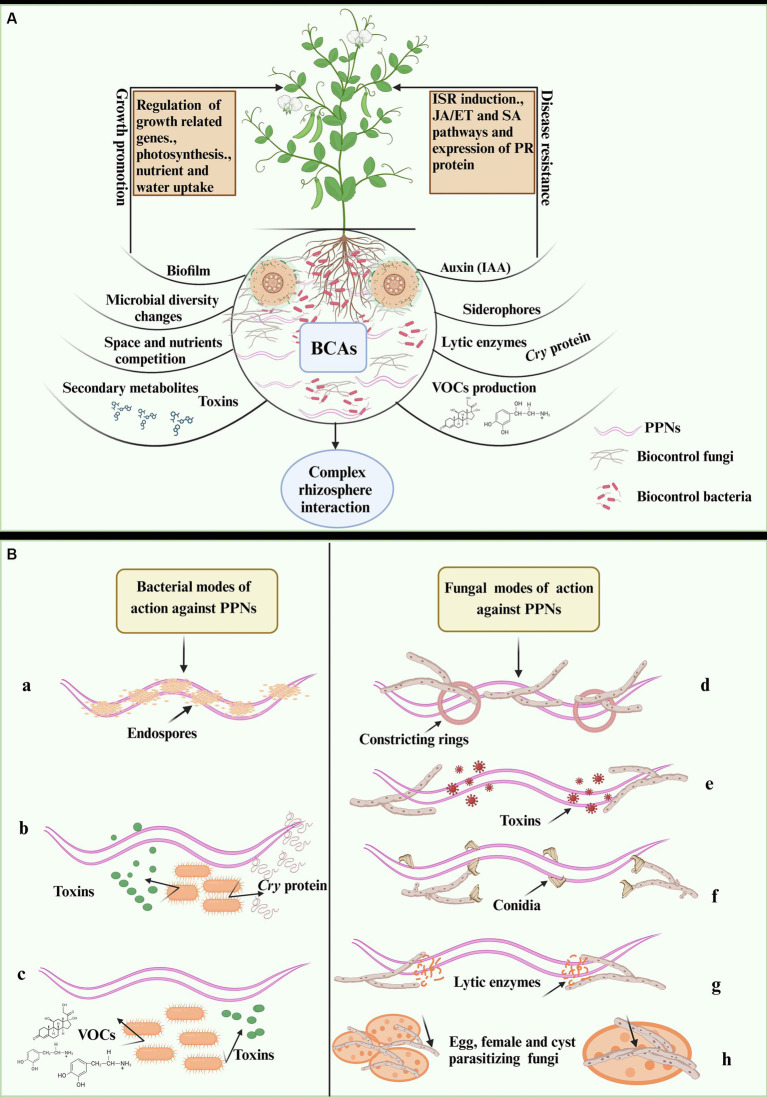
Illustration of bacterial and fungal biocontrol of PPNs **(A)** rhizospheric complex interaction and biocontrol of PPNs in infested plants **(B)** illustration of different modes of bacteria against nematodes (a-c), (a) endoparasitic bacteria (b) crystal forming (opportunistic bacteria) and (c) rhizobacteria. Fungal mode of action against nematodes (d-h), (d) nematode trapping or predatory fungi, (e) toxin-producing fungi (f) endoparasitic fungi, (g) lytic enzymes producing fungi for PPNs suppression and (g) eggs and cysts parasitizing (opportunistic fungi). The figure is designed using biorender https://app.biorender.com dated 4/30/2024.

Bacteria use various mechanisms to suppress PPNs, which can aid in the development of effective biocontrol strategies against nematode infestations. These mechanisms involve direct and indirect antagonistic interactions between bacteria and PPNs (Ayaz et al., 2021, 2023) as illustrated in Figures 1A,B. Studies have shown that bacteria exert direct effects on PPNs through colonization, parasitism, and antibiosis, which include the production of lytic enzymes, antibiotics, toxins, and VOCs (Timper, 2011; Migunova and Sasanelli, 2021). For instance, *Pasteuria* spp., an obligate nematode parasitic bacterium, directly attaches to nematode cuticles and multiplies in nematodes body to destroy them (Orr et al., 2020). Another direct action mechanism involves the production of nematicidal compounds by PGPB such as *Bacillus* spp., which produce extracellular enzymes like proteases, chitinases, collagenases, lipases, and enzyme complexes that affect PPN populations at different life stages (Migunova and Sasanelli, 2021; Pires et al., 2022). Glucanases, cellulases, and pectinases produced in *S. marcescens* were found to control *M. incognita* effectively (Migunova and Sasanelli, 2021; Khan et al., 2023), while crystal (*Cry*) proteins were observed to be produced by *B. thuringiensis* and damage the internal organs in a wide range of PPNs. Additionally, *Bacillus* spp. produce lipopeptides, surfactin, bacillomycin D, fengycins, iturins, and bacteriocins to inhibit *M. incognita* (Penha et al., 2020; Liang et al., 2022).

The compound 2,4-diacetyl phloroglucinol (DAPG) from fluorescent *Pseudomonas* spp. reduces the motility of juvenile nematodes and inhibits eggs hatching of potato cyst nematode, *Globodera rostochiensis*. It has also been reported that endoparasitic nematodes, which live in plant roots and aerial sections, are suppressed by toxins produced by *Photorhabdus* and *Xenorhabdus* species, found in entomopathogenic nematodes (Cronin et al., 1997; Cimen et al., 2022). *Pseudomonas* spp. release hydrogen cyanide, while *Bacillus* spp. produce hydrogen sulfide, both of which have nematicidal effects on important PPNs (Pires et al., 2022). Furthermore, bacterial volatiles like dimethyl disulfide (DMDS) and 2-Undecanone (2-UD) produced by *B. atrophaeus* have shown strong nematicidal activity against *M. incognita* (Ayaz et al., 2021; Ali et al., 2023b). The deep-sea bacterium *Virgibacillus dokdonensis* produces four nematicidal volatiles: acetaldehyde, dimethyl disulfide, ethylbenzene, and 2-butanone. These volatiles exhibit different modes of action against J2 of *M. incognita*, including attraction, repellence, fumigation, egg-hatching inhibition, and direct contact-killing (Chen et al., 2024). Based on past studies, bacteria from various genera contain a wide range of bioactive compounds which need extensive research to explore novel nematicides for sustainable agriculture.

Indirect methods employed by PGPR for PPNs control include siderophore production, hormones, phosphate solubilization, nitrogen fixation, induction of plant ISR, and modifications of the plant microbiome (Ayaz et al., 2021; Migunova and Sasanelli, 2021). PGPR produces indole acetic acid (IAA) to enhance plant growth under nematode infestation and induce host tolerance against PPNs by stimulating the production of various compounds (siderophores, lipopolysaccharides, exopolysaccharides, N-acyl-homoserine lactones, etc.) and enzymes such as ascorbate peroxidase, β-1,3-glucanase, chitinase, catalase, lipoxygenase, phenylalanine ammonia-lyase, polyphenol oxidase and superoxide dismutase (Ayaz et al., 2021; Liang et al., 2022).

The PGPR has also been documented to colonize the plant roots to reduce the severity of PPNs infestation as shown in Figure 1A. Previous studies reported white clover colonization by *B. cereus* B1 and *P. fluorescens* P2 increased host resistance to *H. trifolii.* Further, the combined effect of root colonization by *P. fluorescens* CHA0 and exogenous SA treatment has been found to induce ISR in tomato plants against *M. javanica* (Migunova and Sasanelli, 2021; Pires et al., 2022). Plant bacterial endophytes can be beneficial to their host as they stimulate plant growth and prevent the spread of plant diseases. Tomato roots containing the endophytic bacterium *Bacillus cereus* BCM2 were found to be resistant to J2s of *M. incognita*, and the colonization can significantly minimize damage caused by the nematode (Li et al., 2019). Several studies have been conducted on *Bacillus* as endophytic bacteria for the management of RKN. It has been found that *B. licheniformis*, *B. megaterium*, *B. pumilus*, *B. mycoides*, and *B. thuringiensis* effectively lower the number of galls and egg masses produced by *M. incognita* (Yu et al., 2015). Overall, bacteria from different genera offer a diverse array of bioactive compounds, highlighting the need for developing novel nematicides for sustainable agriculture. The bacterial biocontrol agents and their mechanisms for PPNs control are given in Figure 1B.

### 4.2 Fungal biocontrol mechanisms against PPNs

Utilizing beneficial fungi to control nematode populations in plants and crops is known as “fungal biocontrol of plant nematodes.” Fungi can exert their biocontrol activity through various mechanisms targeting different stages of nematode. Fungi are categorized into different groups based on their mechanisms for suppressing PPNs, as shown in Figure 1B (Jansson and Lopez-Llorca, 2004; Pires et al., 2022). Predatory fungi, such as *Arthrobotrys* and *Dactylella* species use nematodes as their food source by developing specialized structures to capture nematodes. Parasitic fungi such as *Nematophthora* and *Hirsutella*, parasitize nematodes, penetrating and proliferating in their bodies, ultimately causing their death (Migunova and Sasanelli, 2021; Khan et al., 2023). Nematode-trapping fungi, such as *Arthrobotrys* spp. have specialized structures like constricting rings, adhesive nets, or knobs to trap nematodes, immobilizing and consuming them (Pires et al., 2022; Mokrini et al., 2024). Colonizing fungi can colonize plant roots or the rhizosphere, competing with nematodes for nutrients and space, indirectly reducing nematode numbers (Abd-Elgawad and Askary, 2018). Some fungi produce chemicals that prevent nematode eggs from hatching or obstruct their attraction to plant roots. Opportunistic saprotrophic fungi target nonmotile stages, such as nematode eggs, and cysts. *Purpureocillium lilacinum* is best suited to target the nematode eggs, as it can enter the egg through its conidia (Khan and Tanaka, 2023). It has also been reported that fungi, like *Trichoderma* spp. produce secondary metabolites with a potential nematicidal effect. These compounds can either kill nematodes directly or interfere with biological processes, affecting their growth, reproduction, or survival (Jansson and Lopez-Llorca, 2004; Siddiqui and Shaukat, 2004).

Beneficial fungi can also trigger host ISR against PPNs by colonizing plant roots and enhancing plant resistance. Endophytic fungi like arbuscular mycorrhizae fungi (AMF) can enhance nutrient and water uptake during PPN infestations. These fungi regulate plant antioxidant enzymes and defense-related genes, triggering host ISR against PPNs (Schouteden et al., 2015; Poveda et al., 2020; Tolba et al., 2021). Beneficial fungi can activate SA-mediated responses against PPNs, similar to responses induced by necrotrophic pathogens, involving both SA and JA pathways (Pires et al., 2022; Tyśkiewicz et al., 2022). In conclusion, fungal biocontrol agents offer a range of strategies for regulating nematodes through predatory, parasitic, nematicidal, trapping, antagonistic, and defense-inducing mechanisms. These environment-friendly methods provide long-term nematode management in horticultural and agricultural fields, improving soil health, and crop productivity, and reducing reliance on chemical nematicides (Li et al., 2007; Lawal et al., 2022). Further research and development in this field will enhance the capacity of fungal biocontrol agents to combat plant-parasitic nematodes. The different mechanisms involving fungal biocontrol of PPNs are illustrated in Figure 1B.

## 5 Prominent nematicides from bacteria and fungi

Nematicides derived from bacteria and fungi provide environmentally acceptable and sustainable solutions for managing PPNs. These biological control products reduce nematode infestations, promote soil health, and contribute to sustainable crop production (Siddiqui and Shaukat, 2004; Li et al., 2007). Ongoing research and development in this field aim to enhance the application and efficacy of bacterial and fungal nematicides, thereby improving integrated pest management practices and reducing reliance on chemical pesticides (Pires et al., 2022; Khan et al., 2023). This section aims to outline the key bacterial and fungal nematicides highlighted in the literature.

### 5.1 Bacterial-based nematicides

It has been documented that *Bacillus* and *Pseudomonas* spp. along with other rhizosphere bacteria, produce antimicrobial substances that are toxic to nematodes (Li et al., 2015; Ali et al., 2023b). Most research focuses on rhizobacteria lytic enzymes for controlling PPNs. Rhizobacteria produce a variety of lytic enzymes, such as chitinase, protease, cellulase, lipase, glucanases keratinase, etc. These hydrolytic enzymes disrupt nematode biology, development, and metabolism by breaking down the essential chemical components of the nematode exoskeleton and eggshell (Castaneda-Alvarez and Aballay, 2016; Migunova and Sasanelli, 2021). Chitinases hydrolyze the polymeric linkages in the chitin matrix of the eggshell, leading to eggshell rupture and the hatching of immature eggs. Proteases break down the peptide links that hold glucose-protein molecules together, destroying the nematode’s body structure (Kishore et al., 2022). A purified protease from *B. cereus* NJSZ-13 was recently discovered to be nematicidal against the pinewood nematode. It was also observed that nematode vulnerability to nematophagous fungus and antagonistic bacteria, which can lead to the nematode’s death, is caused by the hydrolysis of collagen in the cuticle layer. Collagenase from the strains of *P. fluorescens* FP805PU and *Brevibacterium frigoritolerans* FB37BR strongly affects *M. ethiopica* and *X. index* (Kishore et al., 2022; Li et al., 2023).

Additionally, a lipid layer found in the eggshell of *Heterodera* species of cyst nematodes serves as a protective coat that prevents moisture loss, especially in unfavorable circumstances. The strains of *B. thuringiensis* FB833T and FS213P, as well as *B. amyloliquefaciens* FR203A, exhibit strong lipase activity, making them nematicidal agents against *X. index* (Castaneda-Alvarez et al., 2016). A wide range of other hydrolytic enzymes, such as pectinases, cellulases, and glucanases from *Pseudomonas* spp., influence the amount of *M. incognita* in soil. These lytic enzymes interact with other secondary metabolites to regulate PPNs, highlighting the need for extensive research on their nematicidal mechanisms (Kishore et al., 2022; Ahmed et al., 2023). In addition to lytic enzymes, *B. thuringiensis* produces *crystal protein (Cry) with strong nematicidal activity against PPNs. Several Cry proteins, such as Cry5, Cry6, Cry12, Cry13, Cry14, Cry21, and Cry55* exhibit nematicidal activity by damaging the intestinal lining, affecting pore development, cells, and vacuoles (Li et al., 2015; Liang et al., 2022).

Bacteria also produce potent secondary metabolites with strong nematicidal activity against PPNs. Sphingosine, an unsaturated hydrocarbon chain amino alcohol isolated from *B. cereus* exhibited strong nematicidal activity that caused severe oxidative damage to *M. incognita* (Gao et al., 2016; Kishore et al., 2022). *B. thuringiensis* produces thuringiensin, a thermostable metabolite with strong control efficacy against the soybean cyst nematode (*Heterodera glycines*). Thuringiensin is recognized as an ATP analog that inhibits RNA production by competing with ATP on binding sites, a mechanism like that of aldicarb, a carbamate insecticide. *B. thuringiensis*, the strain that produces thuringiensin, has been shown to produce trans-aconitic acid, a powerful inhibitor of aconitase in the TCA cycle (Jisha et al., 2013; Seong et al., 2021). Volatiles such as 2-undecanone and 2-nonanone produced by *Bacillus* spp. also exhibit strong nematicidal activity against pine wood nematode *B. xylophilus.* The integrity of the nematode pharynx and intestine is destroyed by the 2-nonanone treatment, although more investigation is needed to completely understand the mechanism of action (Deng et al., 2022). *P. fluorescens* produces 2,4-DAPG that functions as a proton ionophore to diffuse the proton gradient across the mitochondrial membrane. The toxicity assay reveals that 2,4-diacetyl phloroglucinol greatly decreases *M. incognita* eggs hatching but has no effect on the juvenile nematodes. Previous studies also reported hydrogen cyanide, a metabolite that certain *Pseudomonas* species generate inhibits mitochondrial cytochrome oxidase and plays a role in the suppression of plant-parasitic nematodes, such as *M. javanica* (Seong et al., 2021; Kishore et al., 2022).

**Figure 2:**
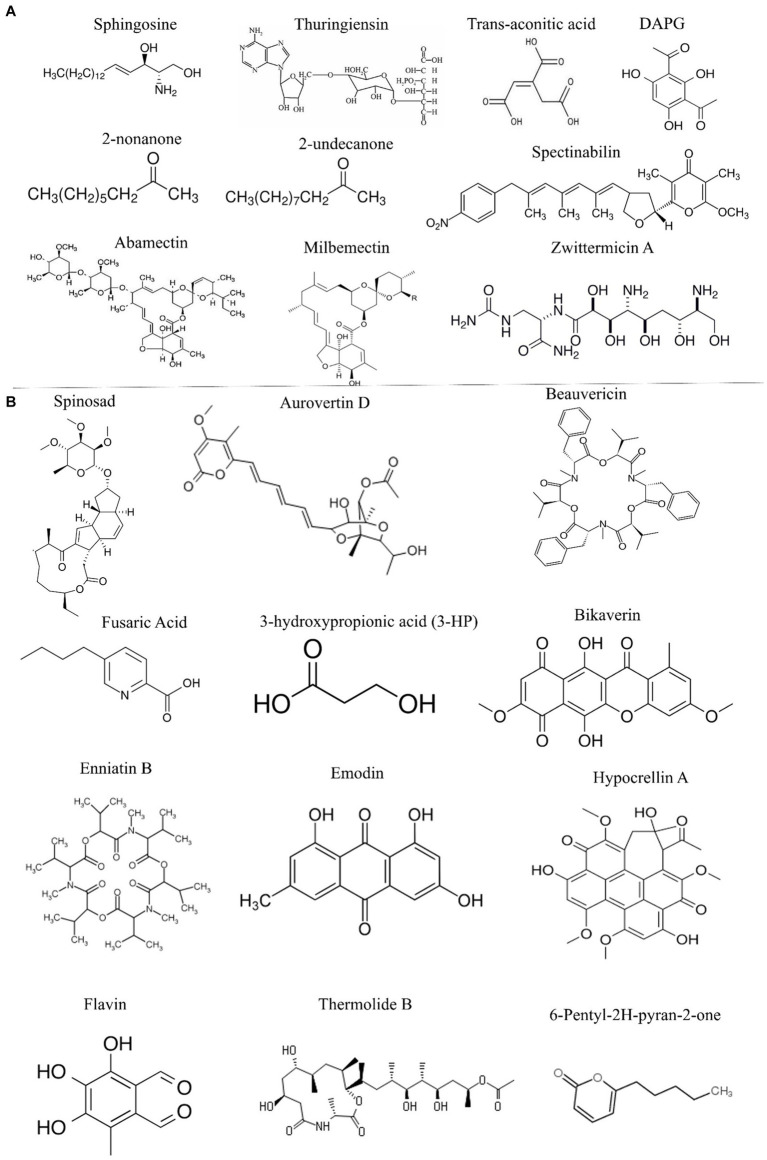
Potential nematicidal compounds from bacterial and fungal biocontrol agents. **(A)** Bionematicides isolated from different bacteria. **(B)** Different bionematicides isolated from fungal biocontrol agents.

Prokaryotic bacteria called actinomycetes are famous for producing a wide range of primary and secondary metabolites with antimicrobial properties against different pathogens (Pires et al., 2022). Avermectin B1, also known as abamectin, is a macrocyclic lactone commonly used to control insects and parasites produced by *Streptomyces avermitilis.* It disrupts glutamate-gated chloride channel receptors, causing high toxicity to plant-parasitic nematodes such as *B. xylophilus* and *M. incognita*. Milbemectin, initially extracted from *S. hygroscopicus*, is comparable to avermectin in terms of its molecular structure and mode of action (Wolstenholme and Rogers, 2005; El-Ashry et al., 2021). Spectinabilin, first identified from *S. spectinabilis* showed considerable nematicidal activity against *B. xylophilus*, with an LC50 value of 0.84 mg/L (Wolstenholme and Rogers, 2005; Seong et al., 2021). Spinosad, a mixture of spinosyns isolated from *Saccharopolyspora spinosa* showed nematicidal activity against *M. incognita*. The compound interferes with gamma-aminobutyric acid receptors and nicotinic acetylcholine receptors in nematodes, disrupting neural activity and subsequent paralysis (Radwan et al., 2019.; Kishore et al., 2022). The identification and function of many microbial secondary metabolites are still unknown, limiting our understanding of the molecular targets of bioactive compounds from PGPR. Due to these limitations, the potential nematicidal effects of certain chemicals are not extensively evaluated in the context of PPNs. Omics and molecular docking approaches will allow us to study these bioactive compounds in a targeted manner to ensure sustainable PPNs control in agriculture. The potential bacterial nematicides documented in the literature are given in Figure 2A.

### 5.2 Fungal-based nematicides

Fungal biocontrol of PPNs provides valuable direction for exploring novel nematicides. Previous studies have identified several fungal species, including *Arthrobotrys*, *Nematoctonus*, *Pleurotus*, *Pochonia* and *Trichoderma*, that exhibit strong nematicidal activity through the production of lytic enzymes, toxins, VOCs, and secondary metabolites (Lawal et al., 2022; Pires et al., 2022). Various compounds extracted from different fungi have shown nematicidal activity against PPNs. For example, Aurovertin D extracted from the nematophagous fungus *Pochonia chlamydosporia* demonstrated a high level of toxicity (LC_50_ value of 16.45 μg/mL) against *M. incognita* (Wang et al., 2015). Phomalactone, a broad-spectrum secondary metabolite isolated from *Nigrospora* spp., has been found to reduce *M. incognita* J2 invasion at a concentration of 500 mg/L in tomato roots (Kim et al., 2001; Kishore et al., 2022). Thermolide from the fungal species *Talaromyces thermophilia* exhibited significant toxicity to *M. incognita* and *B. xylophilus* with LC_50_ values of 0.7–1.0 μg/mL. Hypocrellin A, derived from *Shiraia bambusicola*, caused 50% mortality in *B. xylophilus* at 50 μg/mL (Guo et al., 2012; Seong et al., 2021). Additionally, 3-hydroxypropionic acid (3-HP) extracted from the endophytic fungus *Phomosis phaseoli*, showed specific nematicidal action against *M. incognita*, with an LC_50_ value of 12.5–15 μg/mL (Schwarz et al., 2004). Flavipin from *Chaetomium globosum* inhibits soybean cyst nematodes and root-knot nematodes by blocking ATP synthesis, inhibiting protein synthesis, and enhancing membrane permeability (Madrigal and Melgarejo, 1994; Kishore et al., 2022). Bikaverin and fusaric acid isolated from the endophytic fungus *Fusarium oxygensporum*, inhibited *B. xylophilus* at LC_50_ values of 50 μg/mL and 43 μg/mL, respectively. Although the exact mode of action of fusaric acid is unknown, it has been suggested that fusaric acid might be associated with the chelation of metal ions (Kwon et al., 2007). The depsipeptide beauvericin, produced by *Beauveria bassiana*, had nematicidal activity against *M. incognita*. Its exact mode of action is unknown; however, it has been hypothesized that it may work by activating the mitochondrial death pathway and inducing severe oxidative stress (Mallebrera et al., 2018; Youssef et al., 2020). Beauvericin, a depsipeptide produced by *Beauveria bassiana*, shows nematicidal activity against *M. incognita* possibly by activating the mitochondrial death pathway and inducing severe oxidative stress (Li et al., 2007; Prosperini et al., 2017; Seong et al., 2021). Enniatin B, a cyclodepsipeptide, functions as an ionophore and displays strong toxicity against *M. javanica*, while Emodin, a bioactive quinone from *Aspergillus galucus* displayed potent nematicidal activity against *M. incognita* (Prosperini et al., 2017). Overall, the research on fungal biocontrol of PPNs, coupled with omics and biotechnological approaches, holds promise for developing novel nematicides for sustainable agriculture. The fungal nematicides reported in previous studies are summarized in Figure 2B.

## 6 Linking omics with bacterial and fungal biocontrol of PPNs

In the rhizosphere, plants, and microbes, especially bacteria and fungi, interact to mitigate the effects of pathogen attacks. This interaction involves a complex network mediated by signaling molecules and secondary metabolites from root exudates and microbes in the rhizosphere, attracting the attention of researchers for novel antimicrobial discovery (White et al., 2017; Ahmed et al., 2022; Ayaz et al., 2023). However, the interaction between plants and microbes under PPNs infestation has been rarely studied. Previous studies have mainly focused on microbiome regulation under bacterial and fungal pathogens, with limited knowledge of microbiome and metabolomics regulation for PPNs control (Migunova and Sasanelli, 2021; Cai et al., 2022). Therefore, there is a need to explore potential microbes and their antimicrobial substances in the rhizosphere for PPNs suppression. Here, an effort is made to briefly summarize the importance of omics such as metagenomics, transcriptomics, and proteomics metabolomics along with other biotechnological approaches to explore novel nematicides for efficient PPN control in the future.

### 6.1 Biocontrol of PPNs in metagenomics era

Soil microbes present sustainable opportunities for controlling PPNs in various crops and plants. The rhizosphere harbors a complex structure of soil microbial communities. Metagenomics-based biocontrol of PPNs facilitates the discovery of novel biocontrol agents that are challenging to culture in a laboratory. Next Generation Sequencing (NGS), an innovative technology, has the potential to enhance our understanding of the function and biodiversity of rhizosphere communities. Previous studies have shown that a single gram of soil typically contains 10^3^–10^4^ taxonomic units of microorganisms (White et al., 2017; Kishore et al., 2022; Pires et al., 2022). This indicates that the current ratio of known nematode antagonistic microbes is as small as the total rhizosphere microbial community. Metagenomics based on NGS can help explore potential unknown microbes for nematode suppression, offering novel and sustainable pest management methods in the metagenomics era (Ciancio et al., 2016; Lahlali et al., 2021). Metagenomics provides a comprehensive view of microbial populations by analyzing genetic material directly extracted from environmental samples. This approach aids in identifying new biocontrol agents (BCAs) and comprehending their interactions with PPNs (White et al., 2017; Mohanram Praveen, 2019). The genomics study provides deep insight into the genome of different BCAs and the prediction of genes responsible for plant disease protection. Currently, the genome of the novel bacterium *P. penetrans,* with strong nematicidal activity, is being sequenced. By extracting and sequencing genomic DNA from *Pasteuria* species, such as *P. penetrans,* which cannot be cultured in the lab, researchers can enhance their understanding of its biology and evolution. Comparative genomics studies will provide important insights into the bacterium’s basic biology, especially its role as a nematode pathogen (Marin-Bruzos et al., 2021; Kishore et al., 2022).

Metagenomics research has revealed a broad range of microbes, including bacteria and fungi, in soil and the rhizosphere that can suppress PPNs. Understanding complex microbial interactions within the soil ecosystem is crucial for developing multispecies biocontrol consortia to effectively manage PPNs (Castaneda-Alvarez and Aballay, 2016; Ayaz et al., 2021). Additionally, functional metagenomics allows exploration of silent gene clusters in beneficial microbes that may possess nematicidal activity against PPNs. Cloning and expressing environmental DNA in surrogate host species is a key aspect of functional metagenomics to identify genes producing bioactive compounds or enzymes with nematicidal effects (White et al., 2017; Migunova and Sasanelli, 2021). Manipulating the soil microbiome can enhance the natural biocontrol of PPNs. Targeted microbiome engineering techniques can be developed by gaining in-depth knowledge of novel microbes in the rhizosphere community for PPNs suppression (Syed Ab Rahman et al., 2018; Pires et al., 2022). Integrating metagenomics into biocontrol research revolutionizes sustainable PPNs management. By linking microbial diversity with metagenomics, researchers can explore novel biocontrol agents against PPNs to promote sustainable and resilient farming practices.

### 6.2 Transcriptomics and proteomics for PPNs biocontrol

BCAs and PPNs interact through molecular processes that can be explored using robust omics approaches such as transcriptomics and proteomics. The identification of novel genes, pathways and proteins for PPNs suppression can facilitate the application of targeted biocontrol strategies (White et al., 2017; Yi et al., 2021). Transcriptomic studies can thoroughly determine the gene expression profiles of BCAs during their interaction with PPNs, shedding light on the underlying molecular mechanisms for PPNs control. Differential gene expression analysis of BCAs, whether up-regulated or down-regulated in response to nematodes, can help to identify genes that could enhance the efficacy of BCAs for suppressing different nematodes (Tian et al., 2007; Schouteden et al., 2015). Additionally, transcriptomic research can provide insight into how BCAs respond to environmental stresses that may affect their efficacy against PPNs, such as variations in soil pH, temperature, or moisture content (Crandall et al., 2020).

Proteomic studies enable the identification and measurement of proteins expressed by BCAs during their interaction with PPNs. Differential protein expression analysis offers a more direct assessment of biocontrol activity compared to transcriptomics and can identify important proteins involved in nematode suppression (White et al., 2017; Liang et al., 2022). Proteomics can recognize bioactive proteins produced by BCAs to inhibit nematodes, such as enzymes, nematicidal toxins, or antimicrobial peptides, which can be promising candidates for bionematicide development. Proteomics can also reveal the complex molecular networks underlying nematode suppression by elucidating protein–protein interactions between PPNs and BCAs (Ciancio et al., 2016; Cai et al., 2022). Metaproteomics, a large-scale evaluation of proteins created and/or modified in microbial communities (such as post-translational modifications) has advanced in recent decades. A recently developed technique called metabolite, protein, and lipid extraction (MPLEx) offers ways to extract lipids, polar compounds, and proteins from rhizospheric soil simultaneously, facilitating the discovery of novel proteins and peptides with different modes of action against PPNs from the rhizosphere (White et al., 2017; Cai et al., 2022). In brief, transcriptomics and proteomics provide valuable data for deciphering the molecular complexities of PPNs and BCAs interactions. By using these omics approaches, scientists can uncover and develop more targeted biocontrol strategies for effective PPNs control in the agriculture sector.

### 6.3 Metabolomics-based biocontrol of PPNs

The integration of metabolomics in the biocontrol of PPNs aims to discover potential nematicides derived from complex interactions between plants and microbes in the rhizosphere. Conventional methods for identifying bioactive chemicals against PPNs often fall short, as they rely on cultivating specific microorganisms and extracting metabolites. Additionally, certain rhizosphere microbes are hard to culture in laboratory conditions but are known to produce numerous bioactive compounds against plant pathogens (Topalović et al., 2020; Mokrini et al., 2024). Metabolomics employs advanced techniques such as GC–MS, LC–MS, NMR, and computational tools to explore rhizosphere metabolites in an untargeted manner. The purpose of metabolomics is to analyze an organism’s metabolomes at specific times and under varying conditions (White et al., 2017; Ayaz et al., 2021). Metabolomes consist of various cellular substrates and products from primary and secondary metabolism, playing essential roles in signaling and stress responses, ultimately defining an organism’s chemical phenotype (Horak et al., 2019; Desmedt et al., 2020).

**Figure 3:**
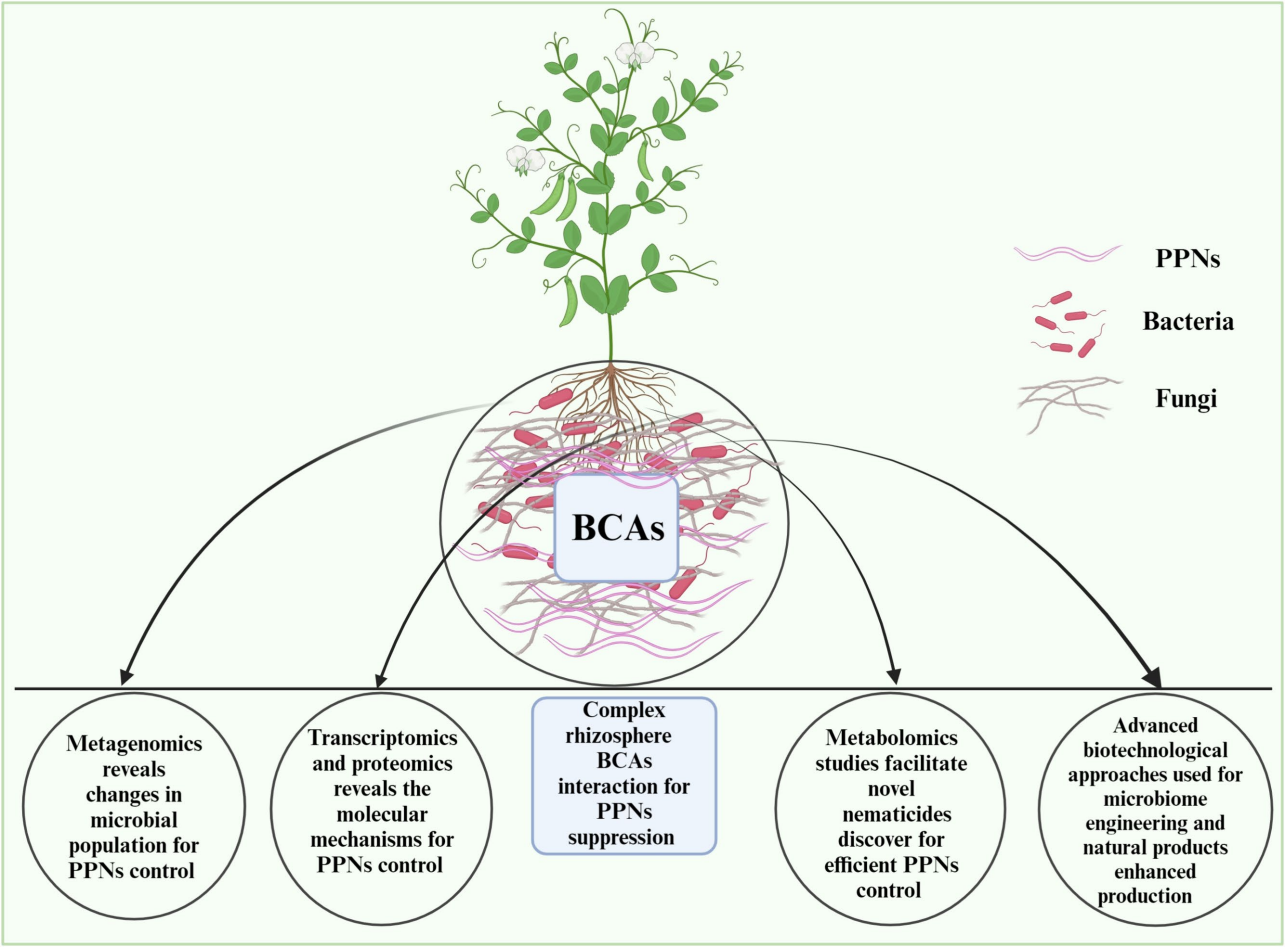
Linking multi-omics approaches such as metagenomics, transcriptomics, proteomics, and metabolomics to improve bacterial and fungal biocontrol of PPNs and novel nematicides discovery. The figure is designed with biorender https://app.biorender.com dated 4/30/2024.

Compared to the transcriptome or proteome, an organism’s metabolome is more sensitive to environmental changes, making metabolomics a valuable tool for understanding the metabolic pathways responsible for phenotypic responses (Bundy et al., 2009). Studies have shown that suppressive soils harbor microbes with strong potential for pathogen control. Metabolic profiling using techniques like NMR and HPLC–MS has revealed differences between conducive and suppressive soils in various agricultural regions (Gómez Expósito et al., 2017; Hayden et al., 2019). For instance, in the context of *R. solani* AG8, untargeted metabolomics approaches have successfully differentiated between conducive and suppressive soils, identifying specific metabolites like lipids, sugars, and terpenes that are more abundant in suppressive soils. Metabolites such as macrocarpals with antibacterial properties have been found to inhibit disease development in these soils (Hayden et al., 2019). In brief, the metabolomics-based research work under biocontrol of PPNs remains unexplored in different plant species. Thus, untargeted metagenomics of various plants rhizosphere metabolome offers a new avenue for exploring novel nematicides. The details of omics approaches needed to improve bacterial and fungal biocontrol of PPNs are shown in Figure 3.

## 7 Challenges in biocontrol of PPNs

The biocontrol of PPNs can be challenging in many ways due to the complexity of nematode biological processes, the diversity of nematode species, and the dynamic interactions among nematodes, plants, microbes and the environment (Cumagun and Moosavi, 2015; Pires et al., 2022). Finding effective BCAs against a wide range of nematode species can be challenging in different environmental conditions. BCAs with strong nematicidal activity against PPNs may show reduced efficacy in field experiments (Schouteden et al., 2015; Pires et al., 2022). Environmental factors such as pH, temperature, moisture content, and soil type can affect the effectiveness of BCAs, requiring their survival in the soil for a prolonged period for sustainable nematode management (Seong et al., 2021; Ayaz et al., 2023). Developing and implementing affordable biocontrol approaches can be challenging due to factors such as high production costs, short formulation shelf life, and inconsistent field efficacy. Obtaining authorization from regulatory bodies and registration for biocontrol products can be expensive and time-consuming (Akhtar et al., 2015; Ayaz et al., 2023). Market adoption of biocontrol products may also be influenced by public acceptance and opinions, requiring education of stakeholders, about the advantages and safety of biocontrol technology through workshops and awareness campaigns. The use of omics technology in the biocontrol of PPNs presents both possibilities and difficulties (Lahlali et al., 2022; Ayaz et al., 2023).

There are certain possibilities and difficulties associated with the biocontrol of PPNs using omics technology. PPNs have complex life cycles with various growth stages and unique interactions with host plants and microbes in the rhizosphere (Abd-Elgawad, 2020; Askary and Abd-Elgawad, 2021). It takes carefully planned studies and time-course analysis to capture the temporal dynamics of metabolite levels, protein abundance, and gene expression. The omics techniques can shed light on the interactions between biocontrol agents and hosts but it is still difficult to maximize biocontrol effectiveness under various field conditions (Kaul et al., 2016; Pires et al., 2022). The integration of environmental factors with BCAs under omics can provide a better understanding, however it makes the data more complex. It takes sophisticated computational methods and bioinformatics knowledge to integrate data from several omics platforms (genomics, transcriptomics, proteomics, and metabolomics) to produce detailed knowledge of PPNs biology and biocontrol mechanisms (White et al., 2017; Sena et al., 2024). In short, overcoming these challenges will open the door to more sustainable and efficient biocontrol methods for managing PPNs in agricultural systems.

## 8 Future perspectives and research directions

Advancements in research and collaboration have opened numerous possibilities for the future of biocontrol in managing PPNs. The utilization of beneficial microbes such as fungi and bacteria as BCAs is steadily increasing (Ayaz et al., 2021; Ali et al., 2023b). Ongoing research focused on identifying and characterizing BCAs will lead to the development of novel and efficient biocontrol products. Biotechnology advances, such as genetic engineering and synthetic biology, have made it feasible to create genetically engineered BCAs that exhibit enhanced effectiveness against PPNs, resulting in the production of targeted nematicides on a larger scale (Pires et al., 2022; Ayaz et al., 2023). The overexpression of genes with nematicidal properties in BCAs offers innovative methods for controlling nematode infestations. Using bioengineering and biotechnology techniques, it may be possible to build BCAs with robust nematicidal activity that can effectively function under diverse field conditions (Abd-Elgawad, 2022).

As climate change continues to impact global agriculture, it will be imperative to develop biocontrol strategies that can adapt to shifting climatic conditions. To ensure long-term sustainability and efficacy against PPNs, research focused on evaluating the performance and adaptability of biocontrol agents under various climate scenarios will be crucial (Abd-Elgawad and Askary, 2020). For the commercialization and adoption of biocontrol technologies, addressing economic viability and negotiating regulatory frameworks will be essential. By increasing public awareness and educating stakeholders about the benefits of biocontrol for sustainable agriculture, acceptance and adoption among farmers, consumers, and legislators can be encouraged (Bonaterra et al., 2022; Ayaz et al., 2023). Training initiatives, educational seminars, and public relations campaigns can aid bridge the knowledge gap and promote the adoption of biocontrol methods for PPNs management. Precision agriculture technology, including drones and remote sensing, can be used to accurately map and monitor nematode infestations at a high resolution. These site-specific biocontrol measures and resource allocation can be optimized for effective nematode management using geographical and temporal data (Sishodia et al., 2020; Arjoune et al., 2022).

The development of microbiome-based biocontrol strategies can be facilitated by understanding the intricate relationship among nematodes, plants, and soil microbes through metagenomics and microbiome research. One potential method of biocontrol is to modify the soil microbiome to promote beneficial microbial communities that can suppress nematode populations (Topalović et al., 2019; Wolfgang et al., 2019). In the future, it may be possible to use an engineered microbiome to regulate the population of nematodes in infested plants. Additionally, bioactive substances with nematode-repellent, nematostatic, or nematicidal properties produced by plants and microbes can be identified quickly with the help of a metabolomics-driven method (Ciancio et al., 2016; Khanna et al., 2021). Despite numerous attempts to collect root exudates using various techniques, distinguishing between plant and microbial metabolites and understanding the outcome of interactions within natural functional networks remains challenging, posing a major obstacle for rhizospheric metabolomics research (White et al., 2017). It is essential to establish optimized methods for accurately sampling and evaluating various rhizosphere fractions. Such optimal methods would address the interconnected nature of plants and microbial metabolic processes in the rhizosphere. Finding bioactive metabolites through screening can result in the development of novel natural products to combat PPNs infestations (White et al., 2017; Verma et al., 2018). Therefore, ongoing advancements in research and omics technology foresee a bright future for the biocontrol of PPNs in the agriculture sector.

## 9 Conclusion

The application of beneficial microbes to manage crop diseases is a safe and eco-friendly strategy that has garnered interest among researchers in crop protection. PPNs pose significant threats to major crop productivity worldwide. This review aims to summarize the status of bacterial and fungal biocontrol strategies for controlling PPNs. It emphasizes the need for further research in areas, such as the development of potential BCAs combined with omics approaches to ensure effective PPNs control in agriculture. The omics era has accelerated the identification, development, and application of fungal and bacterial biocontrol agents for sustainable PPNs management. Utilizing omics can provide a better understanding of the molecular mechanisms involved in biocontrol interactions, enabling researchers to develop effective management strategies for PPNs in agricultural systems.

## Author contributions

MA: Conceptualization, Data curation, Formal analysis, Investigation, Methodology, Software, Visualization, Writing – original draft, Writing – review & editing. J-TZ: Conceptualization, Data curation, Formal analysis, Methodology, Software, Visualization, Writing – original draft, Writing – review & editing. WZ: Data curation, Formal analysis, Investigation, Methodology, Resources, Software, Visualization, Writing – review & editing. Y-KC: Data curation, Formal analysis, Investigation, Methodology, Resources, Software, Visualization, Writing – review & editing. QA: Data curation, Formal analysis, Methodology, Software, Validation, Visualization, Writing – review & editing. FA: Formal analysis, Methodology, Software, Visualization, Writing – review & editing. AK: Data curation, Formal analysis, Methodology, Software, Visualization, Writing – review & editing. QY: Data curation, Formal analysis, Methodology, Software, Visualization, Writing – review & editing. J-WY: Data curation, Formal analysis, Methodology, Software, Visualization, Writing – review & editing. W-CW: Data curation, Formal analysis, Methodology, Software, Validation, Visualization, Writing – review & editing. R-DQ: Conceptualization, Data curation, Formal analysis, Funding acquisition, Investigation, Methodology, Project administration, Resources, Software, Supervision, Validation, Visualization, Writing – original draft, Writing – review & editing. W-KH: Conceptualization, Data curation, Formal analysis, Funding acquisition, Investigation, Methodology, Project administration, Resources, Software, Supervision, Validation, Visualization, Writing – original draft, Writing – review & editing.
